# “I Know How to Identify and Communicate My Needs”: A Qualitative Study of the Self‐Perceived Strengths of People in Polyamorous Relationships

**DOI:** 10.1111/jmft.70119

**Published:** 2026-02-17

**Authors:** Alexander K. Tatum, Sharon M. Flicker, Nawar Albarak, Jessi M. Schroeder, Ash Moomaw, Idil Ugurluoglu, Alex Whitman, Alysse Wiggins, Danielle Davis, Robyn Fisher

**Affiliations:** ^1^ Illinois State University Normal Illinois USA; ^2^ Sacramento State University Sacramento California USA; ^3^ Ball State University Muncie Indiana USA

**Keywords:** consensual non‐monogamy, mononormativity, polyamory, strengths

## Abstract

Little empirical research has adopted a strength‐based approach to examine strategies that help offset challenges (e.g., discrimination, internalized stigma) faced by people in polyamorous relationships. The current qualitative study assessed the self‐perceived strengths of 63 US‐based, adult participants who reported present or former engagement in at least one polyamorous relationship. Participants' responded to the open‐ended question, “What particular characteristics do you have that help you navigate the challenges of polyamory?” A thematic analysis identified strengths across eight broad themes: personality traits, a willingness to challenge mononormative socialization, ability to manage difficult emotions, experiencing compersion and/or low levels of/well‐managed jealousy, strong communication skills, lessons learned from prior relationships, seeking out self‐help and professional resources, and financial privilege. Results provide a roadmap for self‐help and therapeutic approaches to cultivate resilience in individuals who engage in a relationship style that remains heavily stigmatized and can present unique challenges.

## 1 Introduction

Polyamory is a type of consensual non‐monogamy (CNM) in which all involved parties consent to the possibility of multiple loving partnerships (Klesse 2006). In a society where monogamy exists as the prevailing relationship norm, people who practice polyamory experience substantial stigma and discrimination (e.g., Séguin 2019). Yet, research suggests that individuals who practice polyamory cite many benefits to their relationship style (Moors et al. 2017a) and fare as well or better on metrics of relationship quality (e.g., Conley et al. 2017). Identifying the strengths that foster resilience in the face of stigma, discrimination, as well as relationship challenges that may be unique to polyamory, has the potential to inform strengths‐based interventions for individuals engaged in or interested in exploring polyamory. To this end, this study seeks to identify the self‐perceived strengths that help individuals navigate the challenges of polyamory.

### 1.1 Benefits and Challenges of Polyamory

Mononormativity reflects the belief that emotionally and sexually exclusive relationships are ideal. In contrast to monogamous relationships, which are viewed as healthy, natural, and successful, polyamorous (and other CNM) relationships are seen as immoral, unhealthy, unsustainable, and indicative of poor relationship quality (Hutzler et al. 2016). As a result, polyamorous individuals experience rampant stigma from others (Séguin 2019). Polyamorous individuals have reported discrimination from medical and mental healthcare professionals in the forms of non‐inclusive medical paperwork forms, statements that polyamory is sexually unsafe and/or psychologically unhealthy, invasive questioning, and refusal of treatment (e.g., Schechinger et al. 2018; Vaughan et al. 2019). People in polyamorous relationships also face considerable systemic barriers impacting their quality of life, such as legal challenges to their relationships (Morris et al. 2024), fears of losing child custody (Klesse 2019), and an absence of marriage rights (Watson 2022). As a result, many polyamorous people conceal their relationships from others (Füllgrabe and Smith 2023), a strategy that serves a protective function but also can exact a toll on the concealer (e.g., Meyer 2003). Furthermore, people in polyamorous relationships may internalize CNM negativity, which has been shown to affect their well‐being as well as their relationship quality (Moors et al. 2021; Rodrigues et al. 2024).

People in polyamorous relationships may also experience several unique relationship challenges. For instance, while many people in polyamorous relationships do not believe that love is a finite resource to be divided, they may struggle with how time and financial resources are divvied among partners (Codrington and du Plooy 2024; Deri 2015). Furthermore, while jealousy and envy are commonly experienced emotions by individuals in CNM relationships (Deri 2015), these emotions may show up differently in consensually non‐monogamous relationships compared to monogamous relationships. Given consensual agreements that allow intimate engagements with other individuals, jealousy may be experienced not only in relation to fear of losing one's partner to a tempting alternative, but, perhaps more commonly, in relation to fears of losing shared time with a partner or the ability to engage in preferred activities due to the need to balance time and other resources between multiple partners. Additionally, individuals may feel envious of their partner's ability to attract new partners or of the quality of their partner's relationships with others (wishing they also had other relationships of that quality; Flicker et al. 2022).

Despite stigma and other challenges that may be unique to polyamorous relationships (Séguin 2019), people in polyamorous relationships cite many benefits of their relationship structure. Commonly cited benefits include having more people to meet one's needs (both sexual and non‐sexual) and thus having more of one's needs met, engaging in a wider range of activities than a single partner might enjoy, and having the autonomy and opportunity for self‐growth (Moors et al. 2017a; Tatum et al. 2024). Additionally, Conley and Moors (2014) note how the transition from monogamy to polyamory can provide individuals with additional social support. Beyond this, research has consistently demonstrated that individuals in CNM and monogamous relationships report similar levels of relational and sexual satisfaction (Anderson and Norman 2025), undermining claims that those engaged in CNM have low‐quality relationships (Moors and Ramos 2022).

### 1.2 The Present Study

Meyer (2015) advocates for the importance of research into factors that promote resilience among minority populations. Perrin and colleagues (2020) noted that while Meyer (2003)'s minority stress theory has been foundational for studying health outcomes associated holding a minority identity, its deficits‐based focus limits the identification of individual strengths that can promote resilience and consequently positive mental health outcomes and health behaviors. Indeed, prior research has identified multiple possible strengths of those engaged in polyamory. In their review of CNM relationship benefits, Moors and colleagues (2017a) reported that CNM practitioners cited high levels of trust and open communication with their partners. The authors elaborated that CNM relationships can increase social networks, financial resources, and the ability to share household or parenting responsibilities. These outcomes—derived from the social support of a network of partners—can ultimately promote a greater sense of identity pride, self‐esteem, and resilience, akin to how mindfulness may promote adjustment (e.g., greater compersion and less jealousy) through increased emotion regulation skills and distress tolerance (Clemons‐Castaños and Flicker 2025).

Building on what is already known about the benefits of CNM relationships, we sought to identify the self‐perceived strengths of people in polyamorous relationships through a qualitative analysis of individuals' personal reflections on the personal characteristics that help them navigate the challenges on polyamory. Exploring these strengths through the minority strengths model (Perrin et al. 2020) can shed light on the factors that contribute to the well‐being and resilience of individuals involved in polyamorous relationships, as well as broaden the understanding of diverse relationship structures.

## 2 Methods

### 2.1 Participants

The first author recruited participants through the polyamory subreddit (https://www.reddit.com/r/polyamory/) after receiving permission from the forum moderator. Participants were invited to participate in a study “examining the experiences of individuals who have been at least one polyamorous relationship” and were directed to an external Qualtrics survey to complete the study.

Participants met the following inclusion criteria: at least 18 years of age, residing in the United States, and current or previous involvement in at least one polyamorous relationship. A total of 132 responses were recorded in the survey. After removing responses determined to be duplicates or spam, the final sample consisted of 63 participants with an average age of 34.7 years (range = 20–69 years). The sample was largely White (82.5%) with pluralities of women (49.2%) and bisexual (46.0%) participants. Table 1 displays the sample characteristics.

**Table 1:** Summary of participant demographics (*N* = 63).

Demographic	*n* (%)
Age, *M* (SD)	*M* = 34.7 (SD = 9.3)
Gender identity	
Cisgender woman	31 (49.2)
Cisgender man	19 (30.2)
Nonbinary	6 (9.5)
Genderqueer	4 (6.3)
Agender	1 (1.6)
Questioning	1 (1.6)
Transgender man	1 (1.6)
Sexual identity	
Bisexual	29 (46.0)
Heterosexual/straight	11 (17.5)
Queer	6 (9.5)
Pansexual	5 (7.9)
Heteroflexible	4 (6.3)
Lesbian	3 (4.8)
Gay	2 (3.2)
Homoflexible	1 (1.6)
Race	
White	52 (82.5)
Asian/Asian American	3 (4.8)
Black/African American	2 (3.2)
Multiracial	2 (3.2)
Indigenous/American Indian	1 (1.6)
Ethnicity	
Hispanic or Latino/a	8 (12.7)
Not Hispanic or Latino/a	55 (87.3)

### 2.2 Procedure

The study was approved by the first author's Institutional Review Board. Following informed consent, participants responded to six open‐ended questions inquiring about their history and experiences with polyamorous relationships and a demographic survey. These questions were generated by the first two authors and were aimed at broadly understanding participants' experiences in polyamorous relationships. Although the current study focuses on participants' strengths, participants also answered questions related to their motivations for engaging in polyamory, expectations prior to engaging in polyamory, perceived benefits and challenges, and self‐efficacy for managing the challenges of polyamory. Because each question represented a distinct topic and overlap in participants' responses between questions represented a relatively small portion of responses, each question was analyzed and reported separately (Tatum et al. 2024; Tatum et al. 2026). Participants who completed the survey could click a link to access a separate Qualtrics survey where they had the option to enter a raffle to receive one of ten $25 Tango gift cards. Contact information collected in the raffle survey was unlinked with the participants' data.

### 2.3 Data Analysis

The present analysis examines participants' responses to the open‐ended question, “What particular characteristics do you have that help you navigate the challenges of polyamory?” Participants' responses to this question averaged 34 words (range = 4–129 words). The coding team consisted of six individuals. Four of the six coders were doctoral students in counseling psychology, one coder was a master's student in counseling, and one coder was an assistant professor of counseling psychology with expertise in human sexuality and training in qualitative analysis, who oversaw the coding process. The student coders were trained by the first author on the qualitative coding process for this project.

To identify primary themes in the responses, the team utilized Braun and Clarke (2006)'s approach to thematic analysis, which involves allowing themes to arise from the data inductively and progressively through repeated iterations of examining the participants' responses (rather than starting with a pre‐determined set of codes or themes; Kolb 2012). This process involves researchers familiarizing themselves with the data, generating initial codes, combining codes into themes, and continuously reviewing themes until there is consensus on the data. One particularly important aspect of the weekly group coding experiences was the explicit process of bracketing biases (Tufford and Newman 2012). This process seeks to identify the individual coders' biases to avoid making assumptions about participants' intended meaning or experience.

For the current project, two doctoral students on the coding team first independently engaged in an iterative process of reviewing the data, starting by reading through all participants' responses, and generating lists of codes identified from the responses. The second step involved all six team members collectively reviewing and discussing the lists generated by the two doctoral students to identify overlaps in codes between the lists, paring down the number of codes through consensus. Throughout the process, the team repeatedly referred back to the original responses to allow the respondents' voices to maintain primacy. This process resulted in a list of primary and secondary (nested) themes that all six coders agreed represented the data, comprehensively described the main points evident in participants' responses, and were clearly differentiable from one another. Some responses were classified into multiple primary and/or secondary themes. In these instances, the team first discussed whether the responses were better represented by one primary or secondary theme. If the team reached consensus that participants' responses uniquely discussed multiple themes or subthemes, then we classified the response according to primary and secondary subthemes. Throughout the process, differences in interpretation were resolved through discussion at the weekly group coding meetings. The team next revisited the data as a group to identify exemplars for each theme. Finally, the first two authors integrated and consolidated themes where appropriate.

## 3 Results

Primary themes with definitions, secondary themes, and data excerpts that illustrate the primary themes are displayed in Table 2. Below, primary themes are described along with illustrative quotes. Participants' age, gender, and sexual orientation are presented alongside quotes to provide a richer context. It was not uncommon for participants' responses to be classified into more than one primary and/or secondary theme. In these instances, we present the partial data excerpt that best illustrates the given theme. The respective length of each section illustrates the variability between themes in the richness of the data provided by participants. It is important to note that these themes are not viewed as innate strengths of people who practice polyamory; rather, it is clear from participants' responses that many of these strengths often developed though their practice of polyamory and through intensive self‐work.

**Table 2:** Theme identification.

Themes	Subthemes	Data excerpt
*Personality traits* This theme describes “relatively stable, consistent, and enduring internal characteristic[s] that [are] inferred from a pattern of behaviors, attitudes, feelings, and habits in the individual.” (American Psychological Association 2018)	Openness Patience Empathy Pragmatism Independence Flexibility	I'm a patient person and I enjoy spending time alone. –Bisexual woman, 25 years old
*Willingness to challenge mononormative socialization* This theme describes an individual's motivation to push back against cultural messages that assign value to monogamous relationships over polyamorous relationships.		I don't love rules and relationship escalatory things – every relationship I had got re‐negotiated in their own ways. – Straight cisgender man, 46 years old
*Managing difficult emotions* This theme describes strategies used to cope with the experience of unpleasant emotions.	Accountability for one's own emotional experience Distress tolerance (ability to sit with difficult emotions) Emotion regulation strategies	Ability to do in depth self reflection when something is bothering me. I am ok with feeling uncomfortable or sad until I have time to process. – Pansexual cisgender man, 29 years old
*Compersion and jealousy* This theme describes an individual's belief that they possess the capacity to experience happiness for their partner's romantic or sexual connection with others and/or little concern about their partner leaving them for another partner.	Positively valanced emotions observing partner interact with their partners Absent/well‐managed feelings of jealousy	I am not a sexually jealous person and actually enjoy the fact that my partner is intimate with others. –Queer woman, 26 years old
*Communication skills* This theme describes foundational and complex skill sets involving the transparent exchanging of ideas related to the interpersonal relationship dynamic.	Discussing vulnerable emotions Problem solving Proactively identifying and addressing potential problems Listening to partners Transparency/avoidance of indirect communication Creating and respecting boundaries	My partners and I are all very good about communicating our needs before they become larger problems. –Gay trans man, 29 years old
*Relationship experience* This theme describes life experience that is related to having multiple relationships, romantic or otherwise, that allow the individual to develop comfort and skills transferable to polyamorous relationships.	Reflecting on previous relationships Addressing growth edges from previous relationships Applying knowledge from family relationships to romantic relationships	I have learned from the mistakes of my marriage that open and honest communication is the most important part of any relationship, especially when it comes to any topic that feels “difficult” to talk about. –Bisexual woman, 41 years old
*Seeking out self‐help and professional resources* This theme describes material (such as literature and therapy) that can aid adjustment in polyamorous relationships.	Books Online resources Therapy	I am good at reading and researching— found the Secondary's Bill of Rights online. –Nonbinary person, 30 years old
*Financial privilege* This theme describes economic advantages that can ease the challenges of polyamorous relationships.	High income Reasonable/low financial liabilities	Being a higher‐earning professional, which [allows me to] live alone and have a schedule to accommodate multiple partners. – Queer cisgender woman, 40 years old

### 3.1 Personality Traits

Many of the characteristics identified by participants as strengths could be described as personality traits, which the team defined as “relatively stable, consistent, and enduring internal characteristic[s],” (American Psychological Association 2018). Traits were included in this theme if they were referred to by participants as broad internal characteristics that may underpin a variety of specific behaviors, rather than specific behaviors or abilities. The personality traits that aligned with this definition included openness, patience, empathy, independence, pragmatism, and flexibility. Participants noted that these personality characteristics may have prompted their initial attraction to polyamory as well as facilitated resilience in the face of challenges that can be associated with polyamory.

One of the most common personality traits noted by participants was openness, consistent with the Big Five personality trait, Openness to New Experiences (McCrae and Costa 1999):

I'm very open minded, especially regarding the idea of how a relationship ‘should” be.

– Bisexual nonbinary person, 25 years old

Open individuals are defined by their curiosity and interest in challenging traditional ideas. The practice of remaining curious and willing to interrogate what a relationship “should” look like can open the possibility of engaging in relationship structures that more traditional individuals may avoid. Open individuals may also be more creative in problem solving the unique challenges that people who engage in polyamory may experience. Participants also spoke about patience and flexibility:

I have learned how to be more patient and at least not take action until I better understand a situation.

– Pansexual cisgender man, 29 years old

Flexibility and commitment to growth in unexpected situations.

– Heteroflexible cisgender woman, 46 years old

Participants named a handful of other personality traits they believe facilitate adjustment to polyamorous relationships, including an independent nature as well as being empathetic, pragmatic, and confident.

### 3.2 Willingness to Challenge Mononormative Socialization

Mononormative socialization refers to the process of growing up in a society that prioritizes and privileges monogamous relationships. Some participants conscientiously challenged widespread cultural messaging that idealizes monogamy. By pushing back against these norms, participants were able to love others in new ways. For example, one participant wrote:

Previously, I felt that love was finite, it was, in my mind, a resource that could run out. I have come to realize that all loves are different and that they can be incorporated and supported without sacrificing your love for other partners. Even though my partner loves his newer partner deeply, it does not mean that he has diminished love for me.

– Bisexual cisgender woman, 29 years old

This quote sheds light on a commonly held mononormative belief about the scarcity of love, also known as zero‐sum thinking. This belief suggests that a person has a limited quantity of romantic love, and attempts to love more than one person simultaneously results in a “split” of this finite resource (Burleigh et al. 2017), with the recipients receiving less love than they would if they were the sole object of their partner's affection. The above participant described their process of challenging this belief by creating a new narrative that recognizes love as an abundant resource.

The process of challenging monornormative socialization involves the recognition of cultural messages that limit relationships. The following quote provides one such example:

I don't love rules and relationship escalatory things – every relationship I had got re‐negotiated in their own ways.

– Straight cisgender man, 46 years old

This data excerpt references the cultural norm of the “relationship escalator.” Gahran (2017) describes the relationship escalator as “the default bundle of societal expectations for intimate relationships” in which “the goal at the top of the Escalator is to achieve a permanently monogamous relationship. … Escalator milestones include shared ownership of a home, combined finances and having kids together” (pp. 19). The above participant alludes to the relationship escalator's linear, prescriptive, and inflexible tenets that do not permit individuals to deviate from the pre‐established norms. However, this participant described re‐negotiating relationships, allowing those involved in the relationship to choose what their connection will and will not involve. In this way, their relationship agreements may deviate from what would be permitted if he conformed to cultural messages about what romantic relationships are and how they should progress over time.

### 3.3 Managing Difficult Emotions

Participants identified several coping strategies as strengths that helped them navigate difficult emotions, such as jealousy, envy, or anxiety, that arose in their polyamorous relationships. Data excerpts classified under this theme speak to a participant's willingness to take accountability for their emotional experiences, sit with difficult emotions, and employ strategies to regulate distressing emotions:

Ability to do in depth self reflection when something is bothering me. I am ok with feeling uncomfortable or sad until I have time to process.

– Pansexual cisgender man, 29 years old

Ability to take responsibility for my actions and feelings (esp. jealousy, envy, anxiety).

– Bisexual cisgender woman, 28 years old

Instead of stewing on things that are upsetting, I use my words and talk about it.

– Heteroromantic bisexual cisgender man, 38 years old

Understanding that one has control over their emotional experience can help people to manage their responses to difficult emotions, rather than become overwhelmed. Taking accountability for how one responds to one's emotion experience can facilitate working through situations that provoke these difficult emotions with partners and metamours, if appropriate.

### 3.4 Compersion and Jealousy

Compersion includes a range of positive emotions about one's partners' intimate, sexual, and/or romantic involvement with other partners (Deri 2015; Flicker et al. 2021). For participants, the ability to experience positive feelings about a partner's extradyadic relationship means engaging in polyamory can be doubly positively reinforcing: from the joy stemming from their own relationships and from witnessing their partners' intimate relationships with others. Thus, the ability to experience compersion was viewed as a strength that bolstered their relationships. Other participants felt their lack of/well‐managed jealousy was a strength, helping them cope with, and even avoid, relational challenges sparked by jealousy:

I have a lot of compersion – I feel immense joy at a partner's happiness, even if it is not caused by me.

– Bisexual cisgender woman, 41 years old

I'm not particularly jealous which makes it pretty easy to date people who are dating other people. – Bisexual person, 49 years old

I am not a sexually jealous person and actually enjoy the fact that my partner is intimate with others.

– Genderqueer person, 26 years old

Experiencing low levels of jealousy can reduce some of the emotional hurdles that may occur when participants and their partners are involved with multiple people. And while it is possible to experience both jealousy and compersion, low jealousy may be a facilitative condition for the experience of compersion (and vice versa).

### 3.5 Communication Skills

For many participants, the ability to communicate openly and honestly was a core strength underpinning their ability to successfully engage in polyamory.

Poly takes alot of communication.

– Bisexual cisgender woman, 20 years old

I have a love of talking things out, which makes communication easy.

– Lesbian agender person, 24 years old

Because communication is a two‐way street (or more, in polyamorous relationships), participants felt it was important to engage with partners who also were willing to be vulnerable and communicate about their emotions.

Open, honest communication and the willingness to be vulnerable with your partner. If my hubby and I didn't talk constantly about this and bring our feelings to light to be discussed and resolved, I think we would have broken up by now.

– Bisexual cisgender woman, 48 years old

Communication … I never really got holding grudges and not talking, and being passive aggressive and vague. When stuff is off, or I'm annoyed or feel something is wrong I say it and tell them. But I also expect the same from my partner, and we both understand that we need to be open and honest to make sure that we are on the same page.

– Bisexual genderqueer person, 22 years old

The participant described mutual expectations between their partner and themselves that they will share negative emotions with one another in the spirit of transparency. They also provided indicators of maladaptive communication patterns: “not talking,” “being passive aggressive” and “vague” communication.

Other participants identified several concrete strategies that they feel constitutes good communication:

I am a good listener. I know how to create and respect boundaries. I know how to identify and communicate my needs.

– Queer/bisexual cisgender woman, 37 years old

My partners and I are all very good about communicating our needs before they become larger problems.

– Gay transgender man, 29 years old

Both participants above noted the importance of communicating their relationship “needs.” While the former participant identified the importance of listening to being able to communicate well, the latter participant emphasized proactive communication to mitigate potential worsening of the problem.

### 3.6 Relationship Experience

For some participants, experience in previous relationships served as an important foundation for feeling confident in engaging in polyamory today, by providing opportunities to learn from mistakes and develop skills and insights to help them navigate polyamory.

Practice? Years of experience.

‐ Bisexual cisgender woman, 33 years old

I have … learned from the mistakes of my marriage that open and honest communication is the most important part of any relationship, especially when it comes to any topic that feels “difficult” to talk about.

– Bisexual cisgender woman, 41 years old

I have four children and seven grandchildren. I am used to managing multiple relationships and used to the fact that every relationship is different.

– Bisexual cisgender woman, 69 years old

The second participant reflected on their prior romantic relationship as a learning experience for more successful outcomes in their subsequent relationships. Notably, the last participant did not reference romantic relationships but instead identified familial relationships as their basis for appreciating that each relationship is unique and that learning skills to manage multiple relationships.

### 3.7 Seeking Out Self‐Help and Professional Resources

Several participants identified proactively seeking resources that are specifically designed to aid them through anticipated or ongoing challenges associated with polyamory as an important strength. For example:

I am good at reading and researching – found the Secondary's Bill of Rights online.

– Nonbinary person, 30 years old

I had a long time (10+ years) to prepare and do research before my first poly relationship.

– Lesbian cisgender woman, 26 years old

In hierarchical polyamorous arrangements, primary partners are explicitly prioritized in terms of time and other resources, while the role of the secondary partner may be defined by the primary partnership (Kauppi 2021). The Secondary's Bill of Rights is a community‐generated resource that outlines several tenets that honor and respect the dignity of secondary partners in polyamorous relationships. The above participant described this resource as helpful for navigating challenges that arose for them in navigating polyamory. Therapy is another resource that can help individuals navigate relationship challenges, as illustrated by another data excerpt:

I … seek out therapy with polyamorous therapists.

– Queer questioning person, 31 years old

While many kinds of therapy can be helpful, for this participant, it was important for their therapists to have personal identification or at least a professional, affirming understanding of polyamory.

### 3.8 Financial Privilege

Participants spoke about increased access to resources due to their higher socioeconomic status. For instance, participants reported that the ability to live alone facilitated their engagement with polyamory, as illustrated by the following quote:

Being a higher‐earning professional, which [allows me to] live alone and have a schedule to accommodate multiple partners.

– Queer cisgender woman, 40 years old

This participant elaborated that living alone affords greater flexibility to schedule time with multiple partners. Because living alone is typically more expensive than shared housing, higher income facilitates their ability to live alone. The participant above also identified greater ease of scheduling time with multiple partners as a direct function of their income. Polyamorous people who live with a “nesting partner” (i.e., cohabitating partner) may need to negotiate boundaries around when it is acceptable to invite other partners into their home and/or find creative solutions to find space for time with multiple partners. However, a polyamorous person who lives alone does not need to consult with a nesting partner or roommates about the logistics of hosting partners in their physical space.

Another participant extrapolated on the benefits of financial privilege:

I think I'm … situationally privileged in working reasonable hours, having available money and only a medium amount of unpaid care giving responsibilities.

– Mostly lesbian cisgender woman, 30 years old

The lack of extensive paid and unpaid responsibilities provides opportunities to engage in CNM, which can be time intensive, particularly when attempting to manage multiple concurrent relationships. This particular strength requires a degree of professional success or other means of financial independence.

## 4 Discussion

The present study identified factors that promote resilience when faced with challenges stemming from a person's engagement in polyamorous relationships, including personality traits, a willingness to challenge mononormative socialization, an ability to manage difficult emotions, the experience of compersion and/or low degree of jealousy, communication skills, relationship experience, seeking out self‐help and/or professional resources, and financial privilege. Below we consider each of the themes individually.

### 4.1 Personality Traits

Several internal, stable characteristics were identified as associated with resilience when navigating challenges in polyamorous relationships. Openness, characterized by an interest in new ideas and experiences, emerged as one of the most frequently self‐identified personality traits among participants. These findings are consistent with Moors et al. (2017b), who found that Openness to New Experiences is positively related to interest in engaging in CNM among LGB individuals. Individuals high on openness may be willing to challenge traditional ways of relating and are thus able to transcend monogamous relationship norms (Moors et al. 2017b). Polyamorous people characterized by openness may also exhibit greater flexibility and willingness to negotiate and re‐negotiate relationship agreements as new relationships form, or existing relationships change or end.

Other personality traits, such as patience and independence, can serve adaptive purposes when an individual's partner is spending time with their metamour^1^, which has been identified as a more challenging time by some polyamorous individuals (Flicker et al. 2022). Having a fulfilling life outside of one's relationship, consistent with independent personality traits, has been noted as an emotional management strategy for jealousy, envy, and insecurity when one's partner is spending time with another partner (Flicker et al. 2022).

While these personality traits may be innate for some individuals, evidence suggests that individuals have potential to grow in directions that would promote adjustment in polyamorous relationships (Roberts and Mroczek 2008) and participants' responses often reflected the development of strengths through their practice of polyamory and often through intensive self‐work. For example, process research suggests it may be possible to systematically modify habits in order to create lasting, intentional change in personality traits (e.g., Allemand and Flückiger 2017). These findings may establish goalposts for personal work for individuals seeking ways to better cope with the unique challenges that may exist in polyamorous relationships.

### 4.2 Willingness to Challenge Mononormative Socialization

Consistent with previous research (e.g., Codrington and du Plooy 2024; Füllgrabe and Smith 2023), participants in the current study recognized the impact of mononormative socialization on themselves and their relationships and described how challenging these beliefs allowed them to develop more meaningful ways of relating to others. Given the ubiquitous presence of mononormativity, the decision to engage in polyamory requires a willingness to challenge this established norm. For instance, a common belief associated with mononormativity is zero‐sum thinking, or the idea that a person only has a limited amount of love, such that love for one partner necessarily translates to less love for another partner (Burleigh et al. 2017). By pushing back against these socialized beliefs, participants in the current study were able to view love as an abundant quality and feel comfortable about the possibility of their partner loving multiple people.

Internalized negativity, first conceptualized in terms of internalized homophobia (Meyer and Dean 1998) and later broadened to understand the experiences of other minority groups, such as individuals involved in CNM (Moors et al. 2021), is described as the “direction of negative social attitudes toward the self, leading to a devaluation of the self and resultant internal conflicts and poor self‐regard” (Meyer and Dean 1998; p. 161). Internalized CNM negativity is associated with decreased relationship satisfaction and commitment as well as decreased satisfaction with mutually agreed upon relationship and sexual agreements (Moors et al. 2021). In contrast, the ability to extricate oneself from these ingrained expectations can foster resilience in the face of messages that seek to invalidate polyamorous relationships (Séguin 2019). Challenging mononormative beliefs can thus act as a protective factor against internalized CNM negativity, resulting in more positive views of oneself and one's partners (Rodrigues et al. 2024). When grounding these results and prior findings in a minority strengths framework (Perrin et al. 2020), challenging mononormative socialization may ultimately promote mental health and positive health behaviors through the mediating pathways of improving self‐esteem and developing resilience against negative messages about one's polyamorous relationships and/or identity. It may be helpful for therapists to invite clients to reflect on and challenge lingering effects of mononormative socialization as well as identify positive aspects of being part of polyamorous relationships.

### 4.3 Managing Difficult Emotions

Participants’ identification of emotional regulation as a strength is consistent with previous quantitative findings linking higher‐level emotion regulation skills with lower jealousy and greater compersion (Flicker and Sancier‐Barbosa 2024), both of which positively related to relationship satisfaction, as well as qualitative findings (Füllgrabe and Smith 2023): one participant, for instance, acknowledged that, while challenging emotions have not decreased over time, their ability to deal with the emotions have. Emotion regulation is a teachable skill set (Naragon‐Gainey et al. 2017). Learning—and routinely practicing—strategies such as acceptance, tolerating discomfort, mindfulness, and reappraisal may promote resilience and positive mental health described by the minority strengths model (Perrin et al. 2020) as people navigate polyamorous relationships. For example, recent research with polyamorous individuals suggests that mindfulness relates to lower jealousy and greater compersion in response to a partner's New Relationship Energy^2^ with another partner (Clemons‐Castaños and Flicker 2025).

### 4.4 Compersion and Jealousy

Participants noted low levels of jealousy and the ability to feel compersion as strengths that enabled them to effectively navigate polyamory‐related challenges. While some participants in the current study reported being innately predisposed to not experiencing jealousy, others reported increasing ability to cope with jealousy through the development of emotional regulation skills. The attention given to jealousy in polyamorous‐focused self‐help resources (e.g., Chambliss 2017; Labriola 2013) attest to its common experience by polyamorous individuals and these resources espouse a range of strategies to manage it, such as having candid conversations with their partners about underlying insecurities or fears that contribute to jealousy (Deri 2015), self‐care, and emotional processing. Emotion‐focused therapy (Johnson 2019) may be particularly well‐suited for helping clients identify unacknowledged emotions, such as anger or fear, that may be underpinning their experience of jealousy (Kolmes and Witherspoon 2017).

Positive emotions such as compersion have been found to relate to greater relationship satisfaction in participants' relationships with their partner as well as their partners' relationships with participants' metamours (Flicker et al. 2022). It has been proposed that compersion may help lessen the intensity of jealousy (Flicker and Sancier‐Barbosa 2024). However, because unrealistic expectations about jealousy and compersion are occasionally expressed in polyamorous circles and can foster distress and disappointment (Flicker et al. 2022), it is worth noting that neither the presence of compersion nor the absence of jealousy is necessary for healthy or successful polyamory.

### 4.5 Communication Skills

Many participants emphasized their ability to have open, honest, and empathetic communication as an important strength in navigating the complexities of multiple relationships. Effective communication stands as a cornerstone of polyamorous relationships, enabling partners to negotiate agreements, establish clear boundaries, express and honor each other's desires, and offer support, thereby strengthening the bonds within the relationship network. It also underpins effective problem‐solving, which is consistent with previous research suggesting that positive problem‐solving is preferred by individuals in CNM relationships, compared to withdrawal in monogamous relationships (Brooks et al. 2022). In relation to other themes, supportive communication can mitigate conflict through its reciprocal relationship with interpersonal emotion regulation (Nozaki and Gross 2025). Strong communication skills can prevent future relationship problems as well: a meta‐analysis of 64 dyadic longitudinal studies revealed negative communication behaviors (e.g., hostility or defensiveness) were associated with future relationship dissolution (Kanter et al. 2022).

Previous research has identified improvement in communication skills as a benefit of engaging in CNM, resulting in stronger relationships (Codrington and du Plooy 2024) and much of the self‐help literature geared towards polyamory practitioners devote significant space to extolling the importance of open communication and teaching effective communication skills (e.g., Hardy and Easton 2017). Clients may benefit from the frequently used communication skill interventions employed by relationship therapists and may also benefit from reading community‐based resources, such as those promoting non‐violent relational communication (Meenadchi 2023; Rosenberg and Chopra 2015). Therapists can also facilitate the negotiation of formalized relationship agreements between partners, which ideally are formalized as a written document to facilitate periodic revisiting and renegotiating as needed or in response to predetermined criteria (e.g., one partner develops a new connection; Kolmes and Witherspoon 2017).

### 4.6 Relationship Experience

Participants highlighted how past relationships, whether monogamous, polyamorous, or non‐amorous, provided valuable insights into communication styles, conflict resolution strategies, and relationship dynamics, which participants applied to their current polyamorous connections. Insights from previous relationships can foster a sense of relational self‐efficacy that can be carried into future relationships. Following a difficult period in a relationship or a relationship termination, therapists can encourage clients to self‐reflect about their own and their partner(s)' contributions to the difficulty and what one might do differently to better manage or avoid such difficulties in the future.

Conscientious reflection strategies are especially critical due to the lack of relationship paradigms that de‐center mononormative standards. For example, polyamory scholars and practitioners alike have embraced attachment theory as a means of engaging with polyamory (e.g., Fern 2020). While attachment theory may provide a useful framework, it was developed through the lens of a mononormative framework that does not fully encapsulate the nuanced relationships in diverse relationship structures (e.g., metamours). The lack of frameworks and models rooted in polyamorous relationships highlights the need for polyamorous individuals to rely on their own previous relationship experiences as a guide to their present relationships.

### 4.7 Seeking out Self‐Help and Professional Resources

Proactively seeking to learn from educational resources about polyamory was another self‐reported strength. These resources include a wide range of easily accessible, free, online resources in addition to books, workshops, and conferences. Self‐help resources for polyamorous individuals, such as The Ethical Slut (Hardy and Easton 2017) and Polysecure (Fern 2020), include literature written by mental health professionals as well as lay polyamorous individuals and can be used as self‐help materials or within the context of therapy (Kauppi 2021). Therapists can assign bibliotherapy homework assignments (such as chapters relevant to clients' presenting concerns and expressed goals from self‐help books) alongside journaling prompts to facilitate self‐reflection and promote resilience.

Relatedly, the process of connecting with a therapist who identifies as polyamorous or is affirming of polyamory can be important to clients, as some therapists have minimized—or even pathologized—the client's polyamorous relationships (Schechinger et al. 2018; Trexler 2024). To avoid harm that can result from mononormative biases of therapists, therapists should reflect on their own potential biases that may result from the mononormative assumptions prevalent in our society and seek relevant training to prevent these biases from perpetrating harm in the therapeutic context. It is imperative for therapists to educate themselves on best practices for serving polyamorous clients and promoting resilience. Therapists wishing to expand their cultural competency in this area are encouraged to begin by consulting resources distributed by the APA Division 44 CNM Committee (https://www.div44cnm.org/resources).

### 4.8 Financial Privilege

Several participants named financial privilege as facilitating their ability to cope with some of the challenges of polyamory. Individuals with greater financial resources tend to experience more control over their lives. While some polyamorous people report that maintaining multiple relationships can alleviate financial challenges, such as when they live in multi‐partner households and share expenses between them (Sheff 2010), those engaged in solo polyamory, especially those who choose to live on their own, can be aided by financial independence (Falardeau 2022). Living on one's own can make it easier to connect with multiple partners as desired without having to negotiate schedules for the use of shared living spaces. Relatedly, people in polyamorous relationships often name time management as a challenge, driven by the desire to spend time with multiple partners and/or form new intimate connections (Deri 2015). This challenge can be exacerbated by the responsibilities that come with caregiving children or elders or working long hours. Again, while polyamorous people who co‐parent with multiple partners can strategically minimize financial burden by pooling their available resources (Sheff 2010), those with financial privilege may be better able to access paid child‐care and cover other child‐related costs.

## 5 Limitations, Future Directions, and Conclusion

Several limitations of the current study should be acknowledged. First, data were drawn from a sample of U.S. adults who self‐selected into this study while browsing a social media forum geared toward polyamory, limiting the generalizability of the findings in several ways. For instance, this study is missing the voices of polyamorous people who do not seek community based on their polyamorous identity, do not have access to the internet, are not technologically savvy, or prefer not to engage in social media. Additionally, although diverse in terms of age, gender, transgender status, and sexual orientation, the current sample was overwhelmingly White, which mirrors the low representation of Black, Indigenous, and People of Color (BIPOC) in CNM research (Rubin et al. 2014). The absence of this representation speaks to the importance of intentionally centering the voices of BIPOC participants to learn more about protective factors and coping strategies utilized by individuals who may face unique challenges associated with polyamory, including stigma and discrimination based on multiple intersecting marginalized identities (Burns 2021; Pain 2019). Future researchers are encouraged to improve the generalizability of findings by more intentional recruitment of People of Color. To this end, researchers may consider collaborating with social media content creators (e.g., Badiee and Sawyers 2025) to reach audiences less likely to be represented in predominantly White polyamorous spaces or that may be reluctant to engage with academic research.

Perrin and colleagues (2020) acknowledged there are many unexplored strengths‐based pathways to resilience and positive mental health. This study sought to more comprehensively identify self‐perceived strengths of polyamorous individuals. Future research should build on the current findings by examining how these self‐perceived strengths relate to mental health and relationship outcomes and whether they do indeed promote the benefits described by Moors et al. (2017a) in addition to the positive health behaviors described by the minority strengths model.

Additionally, these findings have the potential to facilitate the development of strengths‐based interventions to promote personal well‐being and relationship adjustment for polyamorous individuals. Although some of these identified strengths, such as financial privilege, may be beyond the scope of therapeutic change, the current results provide a roadmap for the development of self‐help and therapeutic interventions to empower people engaged in polyamory to cultivate resilience in the face of common challenges that may arise polyamorous relationships. Therapists can help clients accept accountability for their own emotional experiences, develop effective emotion regulation strategies, become more comfortable with open communication, and process previous relationship experiences for lessons to be applied to future relationships. Testing the effectiveness of these interventions in randomized control trials in accordance with Perrin and colleagues' (2020) minority strengths model would equip clinicians working with people in polyamorous relationships with concrete interventions to aid client growth.

## Data Availability

Researchers may contact the first author to obtain de‐identified data.

## Endnotes

1 A metamour is the intimate partner of one's partner, typically with whom one is not intimately involved themselves.

2 New Relationship Energy is the sense of infatuation one may experience with new romantic, intimate, and/or sexual partners (Stewart 2001).
